# Intraluminal Duodenal Diverticulum: A Rare Cause of Chronic Epigastric Pain

**DOI:** 10.1055/s-0040-1712541

**Published:** 2020-06-16

**Authors:** Shadi Nassar, Alaa El-Kheir, Charif Khaled, Anis Nassar, Joseph Boujaoude, Cyril Tohme

**Affiliations:** 1 Department of General Surgery, Faculty of Medical Sciences, Lebanese University, Beirut, Lebanon; 2 Department of Diagnostic Radiology, University Medical Center, Lebanese Hospital Geitaoui, Beirut, Lebanon; 3 Department of Gastroenterology, University Medical Center, Lebanese Hospital Geitaoui, Beirut, Lebanon; 4 Department of General Surgery, University Medical Center, Lebanese Hospital Geitaoui, Beirut, Lebanon

**Keywords:** diverticulum, duodenal diverticulum, chronic epigastric pain, nausea and vomiting

## Abstract

Despite its first identification in 1885, intraluminal duodenal diverticulum remains a rare entity and only a few case reports are found in the literature. Its diagnosis is almost always delayed due to the lack of specific symptoms and to the very vague presentation consisting of mild epigastric discomfort. However, with the aid of new diagnostic modalities and imaging, it has become easier to diagnose this entity when its symptoms persist. Finally, it can remain undiagnosed in asymptomatic patients.

## Case

We present a case of 21-year-old female patient with a negative medical and surgical history. She presented to the gastroenterology clinic for chronic epigastric pain for several months, associated with postprandial episodes of nausea and vomiting. Her physical examination was insignificant. She was prescribed proton pump inhibitors and prokinetics.

**Fig. 1:**
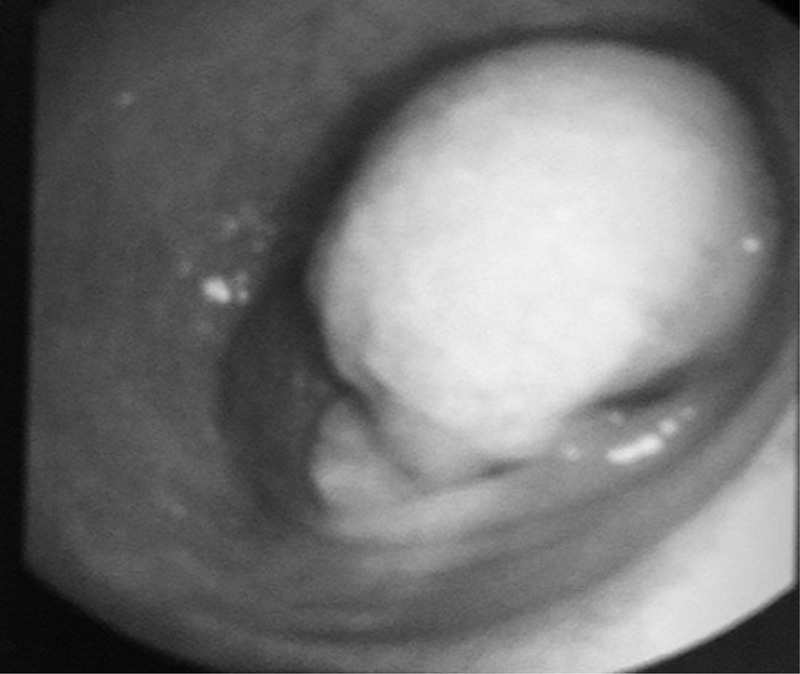
Gastroscopy showing bulging of the second segment of the duodenum.

**Fig. 2:**
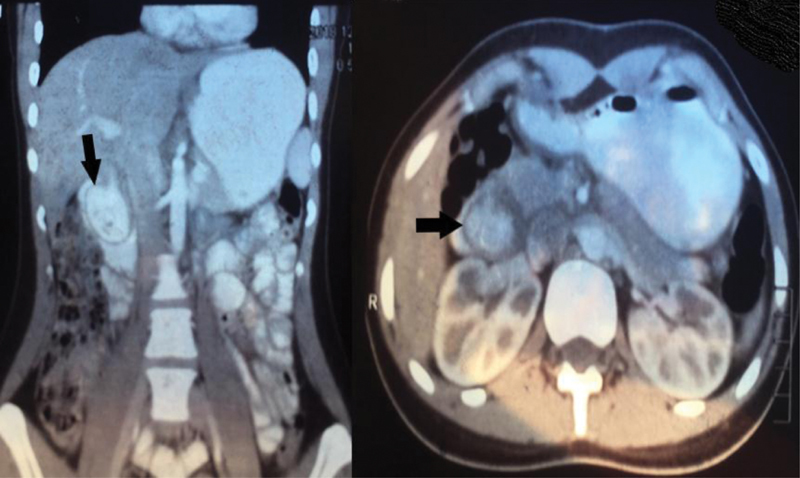
CT scan of the abdomen and pelvis, showing the duodenal diverticulum obstructing the lumen of the duodenum (black arrows). CT, computed tomography.

However, her symptoms didn't improve much, so a diagnostic upper esophagogastroduodenoscopy was done and showed normal mucosa but with external compression on the second part of the duodenum ( Fig. 1 ). Computed tomography (CT) scan of the abdomen and pelvis was ordered due to characterize the external compressing mass. It showed a diverticulum at the second part of the duodenum (D2) measuring approximately 41 mm × 28 mm, filling the duodenal lumen (intraluminal diverticulum) and obstructing it ( Fig. 2 ). Finally, an endoscopic ultrasound was done to delineate the diverticulum and have a clearer view of the surrounding structures. It showed again the intraluminal duodenal diverticulum (IDD) in D2, with a thin and regular wall. The endoscopic ultrasound also confirmed normal pancreatic appearance and normal Wirsung's duct and biliary tract.

The patient was presented with her options and after a surgical consult, and due to her intractable symptoms, it was decided to go with surgical treatment. Written consent was taken from the patient. She was prepared for the operating room.

Intraoperatively, the duodenum was first completely exposed. Then, an intraluminal mass was palpated at the level of D2. Through a longitudinal duodenotomy, the intraluminal diverticulum was identified. The diverticulum was identified 20-mm proximal to the papilla. The papilla was intubated to avoid injury during traction of the mucosa. Excision of the diverticulum was then performed and the mucosa was approximated using absorbable sutures.

The postoperative course was unremarkable and the patient was discharged on day 6 after regaining bowel movement and tolerating oral intake. During her follow-up in the outpatient department, a week after her discharge, she reported complete resolution of her preoperative debilitating symptoms of pain and nausea.

## Literature Review

By definition, a diverticulum is a sac-like protrusion that can occur through gastrointestinal (GI) tract. Diverticula of the small bowel are very uncommon and tend to be asymptomatic. They are usually found incidentally on imaging. Approximately 2 to 5% of patients undergoing upper GI barium studies and 7% of patients undergoing endoscopic retrograde cholangiopancreatography (ERCP) have been reported to have duodenal diverticula. Diverticula are found in most cases in the second portion of the duodenum. 1 2

According to the shape and the etiology of the diverticula, they are classified as extraluminal duodenal diverticulum (EDD) or IDD.

EDD are acquired and consist of an outpouching of mucosal and submucosal layers that herniate through a muscular defect in the bowel wall. The precise mechanism for EDD development remains unknown. However, some theories suggest the presence of a low resistance area in the bowel wall, which may have been created by the passage of the biliary duct, pancreatic duct, or blood vessels through duodenal wall. Another theory proposes congenital causes like the absence of an adequate muscle coat or heterotrophic pancreatic tissue. 1

IDD or “windsock” diverticulum is a protrusion of the mucosa into the lumen of the duodenum. It is a rare congenital abnormality that is probably due to an incomplete recanalization of the foregut lumen. 1 2 It develops between the fourth and eighth embryologic week. Incomplete recanalization may result in a duodenal diaphragm. With time, and due to lack of innervation and peristaltic wave, this diaphragm increases to two or three times of its original size. 2 3

IDD is actually becoming more and more common in adults due to the increased use of radiologic and endoscopic investigations. The reported frequency is as high as 22% at time of autopsy. Note that, less than 10% of duodenal diverticula are actually symptomatic with less than 1% requiring definitive treatment.

The median age of diagnosis is between 30 and 39 years, but it can also be found in pediatric ages. 3 The majority of cases are asymptomatic; however, when symptoms appear, they can range from simple abdominal discomfort and postprandial epigastric pain (90% of the cases), to an upper gastrointestinal obstruction due to the mechanical obstruction by the intraluminal protrusion of the diverticulum. It can even lead to weight loss if not treated early. Symptoms can also present as GI bleeding which can cause chronic anemia. Rarely patients present with massive upper GI bleeding. Some patients also suffer from pancreatitis due to external obstruction of the papilla from the distal end of the diverticulum which is not attached to the duodenal wall, or by reflux of duodenal contents into the pancreatic duct resulting from change of hydrostatic balance. 2 3

Diagnosis of IDD is difficult upon presentation. Imaging is always necessary to confirm the diagnosis. Around half of them (55%) are seen on upper GI contrast studies. Nevertheless, CT remains more sensitive because the endoscopy can report normal mucosa and normal duodenal appearance in cases of IDD; whereas CT scan can easily identify a globular out-pouching in the duodenum. 1 2 4 Endoscopic ultrasound is not routinely used for the diagnosis and management of duodenal diverticula.

Surgical treatment of asymptomatic duodenal diverticulum is not indicated because there is no risk of malignant transformation. Intervention is only required for symptomatic patients who are nonresponsive to medical treatment, or who develop complications such as bowel obstruction, GI bleeding, or pancreatitis. Historically, gastrojejunostomy was performed for gastric outlet obstruction, prior to the identification of the IDD. Nowadays, excision of the diverticulum has become the treatment of choice. The excision can be performed endoscopically (first performed in 1979), or surgically via an antimesenteric duodenotomy after identification of the ampulla of Vater. 4 In 2013, Meinke et al described the first laparoscopic excision of an IDD. It had excellent results and the advantage of a faster and better postoperative recovery over the open technique.
